# Effectiveness of a starch thickened infant formula with reduced lactose content, probiotics and prebiotics on quality of life and clinical outcome in infants with regurgitation and/or colic

**DOI:** 10.3389/fnut.2023.1164722

**Published:** 2023-05-25

**Authors:** Jean-Pierre Chouraqui, Sandra Brancato, Berenice Delmas, Thierry Hanh

**Affiliations:** ^1^Pediatric Gastroenterology and Nutrition, University Hospital Grenoble-Alpes, La Tronche, France; ^2^Association Française de Pédiatrie Ambulatoire (AFPA), Brignon, France; ^3^Département Médical Nutrition Infantile, Nestlé France, Issy-les-Moulineaux, France

**Keywords:** functional gastro-intestinal disorders, infants, regurgitation, colic, comfort formula, thickened formula, *Limosilactobacillus reuteri*

## Abstract

**Background:**

Regurgitation and colic are quite common in young infants, leading to a reduced quality of life (QoL) and to parental distress. Their management is challenging and aims to effectively reassure and relieve symptoms. This study aimed to assess the effectiveness over 30 days of a starch thickened formula with a reduced lactose content, *Limosilactobacillus reuteri* (*Lactobacillus reuteri*) DSM 17938 and FOS/GOS.

**Methods:**

A real-world prospective multicenter experimental study was conducted in a before-after design within subject. Full term infants 0−5 months with regurgitation or colic or both symptoms and without intercurrent illness were included after parental informed consent and received the studied formula. The primary endpoint was the improvement in QoL using the QUALIN infant’s questionnaire. Secondary endpoints were the symptoms outcome and the formula tolerance.

**Results:**

Of the 101 infants included (age: 6.2 ± 4.3  weeks), 33 had regurgitation, 34 colic and 34 had both. At D30, the QoL score was improved in 75% of infants in per protocol analysis (*n* = 68; +8.2 ± 13.7; *p* < 0.001), more in those with colic or both symptoms. Meanwhile, in intention to treat analysis (all *p* < 0.001), the daily number of regurgitations decreased by 61% and the weekly number of days with colic by 63% while the daily cumulative duration of crying decreased by 82 ± 106 mn. These improvements were observed within the first week by 89 and 76% of parents, respectively.

**Conclusion:**

The study formula associated with reassurance is shown to be quickly effective in the management of infant’s regurgitation or/and colic in routine clinical practice.

**Clinical trial registration:**

https://clinicaltrials.gov/, identifier NCT04462640.

## Introduction

Functional gastrointestinal disorders (FGIDs) are frequent in otherwise healthy infants and include a variable combination of recurrent or chronic symptoms (1). The global prevalence of at least one FGID in infants has been estimated between 25% and 30% in European studies (2, 3) and above 50% in Italian infants under 6 months (4). Regurgitation, and colic are the most frequent FGIDs in infants and often coexist (2, 3, 5–7). The pathophysiology of these symptoms is far from being able to be explained whereas they should naturally resolve over time (1). Their respective prevalence varies among the different studies due to differences in diagnostic criteria, study design, data collection methods, cultural habits, availability of health centers, and diet (7). The formal consensus diagnosis of these disorders relies on the symptom-based Rome criteria that have evolved over time until the latest ones established in 2016 as the Rome IV criteria (1). In infants 0–6 months, a recent review reported a prevalence of colic of 10%–15%, of regurgitation of 34% and constipation of 1.5% according either to Rome III or Rome IV criteria (7). In France, according to two studies using Rome III and Rome IV criteria, the respective prevalence of regurgitation, colic, and constipation in infants have been estimated 17%−41%, 18%−19%, and 6%−9% (2, 8). Frequently infants may have multiple FGIDs, notably both colic and regurgitation (3, 9, 10). Formula-fed infants are more likely to suffer from FGIDs, mainly functional constipation, than breastfed infants (3, 9, 10).

Frequent regurgitation and unexplained and inconsolable crying display distressing and anxiety-provoking for parents, driving them to seek frequent medical advice (5, 11–14). Parents are all the more worried, desperate and demanding when the infant display a reduced quality of life (QoL) (5, 9, 13, 15, 16). The Rome IV consensus statement stipulate that physicians should be aware of the impact of the FGIDs symptoms on the infant’s QoL in addition to their clinical assessment (1).

Parents are understandably eager for a quick and easy fix and will often opt for medication in hopes of quick symptom relief (17). Clinicians depend on the reports and interpretation of the parents regarding the symptoms and must meet their expectations. Together, this leads to numerous changes in infant formula, the use of over-the-counter medications, an over-prescription of drugs despite recommendations regarding their uselessness, and therefore an increase in healthcare costs (13, 15, 18). In particular, despite their lack of efficacy in this indication, proton pump inhibitors are increasingly used in infants presenting with unexplained regurgitation or crying, restlessness and irritability that define colic (19–22).

The natural history of infant colic and regurgitation is a spontaneous gradual improvement from the age of 4 and 6 months, respectively (3, 9, 10). In the meantime, the management goals are to provide effective reassurance and symptom relief without requiring medication (1). Therefore, conservative measures such as a dietary approach and/or the use of probiotics are attractive as a first-line management of these common FGIDs (12). Both measures are the most frequently prescribed by French practitioners (8, 9, 15). According to the report of the parents of the 8,865 French infants aged 2 months included in the ELFE study, either a thickened formula, or a thickened formula plus pre- and/or probiotics or a regular formula enriched with pre- and/or probiotics were, respectively, used in 9, 44, and 17% of infants with regurgitation (*n* = 1,098; 12.4%) and 6, 37, and 26% of infants with colic (*n* = 1,921; 21.7%) (23). The use of a thickened formula is the optimal initial management of uncomplicated regurgitation recommended by the joint committee of the North American and the European Societies for Pediatric Gastroenterology, Hepatology, and Nutrition (NASPGHAN/ESPGHAN) and in the National Institute for Health and Care Excellence (NICE) guideline (24, 25). The management of infants with colic may be more challenging insofar as the level of evidence of the different approach proposed is low (26–30). The strongest evidence for the treatment of infantile colic is with probiotics, i.e., live microorganisms that, when administered in adequate amounts, confer a health benefit on the host (31), primarily probiotic strains of *Limosilactobacillus reuteri* previously named *Lactobacillus reuteri* (26, 30, 32, 33). Several randomized controlled trials found that, *Limosilactobacillus reuteri* DSM 17938 (LrD), can reduce crying and/or fussing time in breastfed infants with colic whereas the results of the scarce studies in formula-fed infants are contradictory as reported in systematic reviews and meta-analysis (16, 30, 32, 34–37). The Rome IV recommendations already concluded on the “need for prospective studies to show the efficacy of different diets in infants with FGIDs” leading to new studies considering the specific composition of the formula (1).

The current study aimed at assessing the effectiveness in routine clinical practice of a thickened formula with reduced lactose content and supplemented with LrD and a prebiotic mixture of fructo-oligosaccharides (FOS) and galacto-oligosaccharides (GOS) on QoL and on the symptom relief in infants with regurgitation and/or colic. As the probiotics, the prebiotic mixture was thus considered according to the definition set by the International Scientific Association for Probiotics and Prebiotics (ISAPP) (31).

## Methods

### Study design

This open real-world prospective multicenter experimental study was conducted to assess the effectiveness of the study formula over 30 days in exclusively formula-fed infants 0–5 months with regurgitation or colic or both. The composition of the formula used is detailed in Table 1 in accordance with EFSA recommendations and the European Commission Delegated Regulation 2016/127 (38, 39). The study formula contained *L. reuteri* DSM 17938 at concentration that guarantee a daily intake of approximately 10^8^ colony-forming unit (CFU). The method used a pretest-posttest within-subjects design.

**Table 1:** Composition of the study infant formula per 100 mL and 100 kJ.

	Study formula		EFSA recommendations (38)
	/100 mL	/100 kJ	
Energy	67 kcal (280.3 kJ)	100 kJ	250–293 kJ/100 mL
Protein	1.2 g	0.43 g	0.43–0.60 g /100 kJ
Casein	0.36 (30%)	0.13 g	
Whey protein	0.84 (70%)	0.30 g	
Total fat	3.6 g	1.3 g	1.1–1.4 g/100 kJ
DHA^a^	16.8 mg	6 mg	4.8–12 mg/100 kJ
ARA^b^	16.8 mg	6 mg	
Carbohydrates	7.2 g	2.53 g	2.2–3.3 g/100 kJ
Lactose	5.2 g	1.84 g	≥1.1 g/100 kJ
Starch (95% potato, 5% rice)	2 g		≤2/100 mL
FOS^c^/GOS^d^	0.04/0.36 g		
Magnesium	6.2 mg	2.2 mg	≥1.2 mg/100 kJ
Osmolarity	21.1 m0sm		

a DHA, docosahexaenoic acid.

b ARA, arachidonic acid.

c FOS, fructo-oligosaccharides.

d GOS, galacto-oligosaccharides.

The study protocol, the parents’ information sheet and the informed consent form were reviewed and approved by the Subject Protection Review Board “Sud Mediterrannée III” (Nimes 2020/03/18, no. 2020.01.07 bis_19.12.26.60314). The study was registered in the Clinical Trials Protocol Registration System at ClinicalTrials.gov with the identifier NCT04462640. It was conducted in full agreement with the guidelines laid down in the Declaration of Helsinki and the French data protection act (*Loi* “*Informatique et Libertés*”), ensuring that respondents’ personal identity is withheld. The study was carried out in collaboration with a group of family paediatricians.

### Participants

The parents consulting for regurgitation and/or colic in their exclusively formula-fed infant were invited to participate in the study by their pediatrician, provided that they had a sufficient French language competency and that they were able to use a computer.

Otherwise, healthy infants under six months whose parents were worried about frequent regurgitations or/and crying, irritability, and fussing that start and stop without obvious cause, which are considered as colic in routine practice, were eligible for inclusion. They did not have to fulfill Rome IV criteria (1) since this was a real-world experimental study. They must also have been born at term (≥37 weeks of gestation) with a birth weight ≥2,500 g. The exclusion criteria were: intercurrent acute or chronic illness including suspected or confirmed food allergy; current drug treatment or food supplement other than vitamins; feeding with a partially or extensively hydrolyzed protein formula; failure to thrive. All this information was known to the pediatrician who the infant’s usual doctor was. Each paediatrician had to include all consecutive infants fulfilling inclusion and exclusion criteria and was asked to include, as much as possible, the same number of children with regurgitation or colic. After offering a fully informed description of the survey, parents gave their consent to participate, without any financial incentive.

### Course of the study

The initial consultation (D0) consisted of verifying that the infant met the above inclusion criteria, examining the infant and obtaining the parental informed consent. The paediatricians had to collect anamnestic data and to assess the health status of the infant. Baseline data included date of birth and of the visit, gestational age, gender, birth and current weight and length, number of children in the family, current and previous type of feeding, number and volume of daily feeding in the previous 3 days, previous treatment, and the detailed description of the FGIDs symptoms. These data were reported by the practitioners in an online specific clinical chart derived from the one developed by the Rome Foundation (40). Parents were asked to complete the QoL questionnaire, that was the validated QUALIN questionnaire specifically designed for infants (41). At the end of the visit, parents were asked to move their infant feeding to the study formula for one month.

One month (±3 days) after inclusion (D30), the same charts were completed by the paediatricians and the parents, respectively. Data concerning the course of FGIDs, as well as the efficacy and tolerance of the prescribed infant formula, including the possible adverse effects, were recorded. Parents also had to testify to the doctor about their degree of satisfaction.

All data (D0 and D30) were reported by the physician on an electronic patient reported outcomes software (DACIMA ePRO, Montreal, Quebec, Canada). Data were collected and analyzed by a Clinical Research Organization (CRO, Keyrus Life Science, Levallois Perret, France).

### Data and statistical analysis

The primary endpoint was the outcome of the infants QoL as assessed by parents. Secondary endpoints were the outcome of the symptoms and anthropometric data as well as the tolerance of the study formula and the parents’ satisfaction assessment.

Sample size was calculated with respect to the primary outcome and according to the results of a previous study leading to expect an improvement of 13.5 points in the QoL score with a standard deviation (SD) of 20 (15). Also considering a correlation coefficient of 0.5 between QoL scores at D0 and D30 with an α level of 0.05 and a power (1 − β) of 0.95 and assuming a dropout rate up to 40%, we calculated that 90 infants equally divided between regurgitation and colic should be included.

The QUALIN questionnaire (Supplementary File) includes 34 items with 6 possible answers, which were definitely false, mostly false, both true and false, mostly true, definitely true and do not know (41). The answers were scored from −2 (definitely false) to +2 (definitely true) when it is a positive question, whereas negative items are reverse-scored, from −2 “definitely true” to +2 “definitely false.” As a result, the overall score might range from −68 (worst QoL) and + 68 (best QoL). Following the results of the principal component analysis performed during the validation study, some answers have been grouped in four topics, namely behavior and communication (items no. 3, 5, 8, 13, 16, 18, 21, 22, 24, 32, 33), ability to remain alone (items no. 7, 10, 15, 17, 23), family environment (items no. 11, 26, 29, 31), and psychological and somatic well-being (items no. 4, 6, 14, 19, 27, 30), leaving 8 items out (items no. 1, 2, 9, 12, 20, 25, 28, 34) (41).

All reasons for dropping out including lost to follow-up, stopping or not taking the formula and/or adding a drug treatment, and all adverse effects had to be collected. The analysis of primary outcome was performed on the per protocol population (PP), i.e., the infants who completed the study without violation of the protocol and for which the QoL questionnaire was completed at D0 and D30. The analyses of secondary outcomes were performed first on the intention to treat population (ITT), i.e., all infants included at D0 who were not lost to follow-up and therefore have consulted again at D30, and second on per protocol population.

At each visit the symptoms were assessed according to their frequency and characteristics during the week prior to the visit as reported by parents. Dichotomous variables were described as numbers and percentages, and continuous variables by the mean ± SD and by median, interquartile range (*Q*1–*Q*3) and range (minimum-maximum) listed in brackets. The assessment of stool consistency was adapted from the Bristol stool form scale (42).

Statistical analyses were done using SAS software version 9.4 (SAS Institute Inc., Cary, NC, United States). Means of quantitative variables were compared using the Student’s *t*-test for paired normally distributed data, otherwise the paired non-parametric Wilcoxon signed-rank test was used. Linear regression was used to test independence between the total QoL score and the evolution of symptoms (number of regurgitations/day and number of days with crying) and the Pearson correlation coefficient was calculated. The two-sided alpha level of significance was set at 5%.

## Results

### Participant flow and baseline characteristics

**Figure 1:**
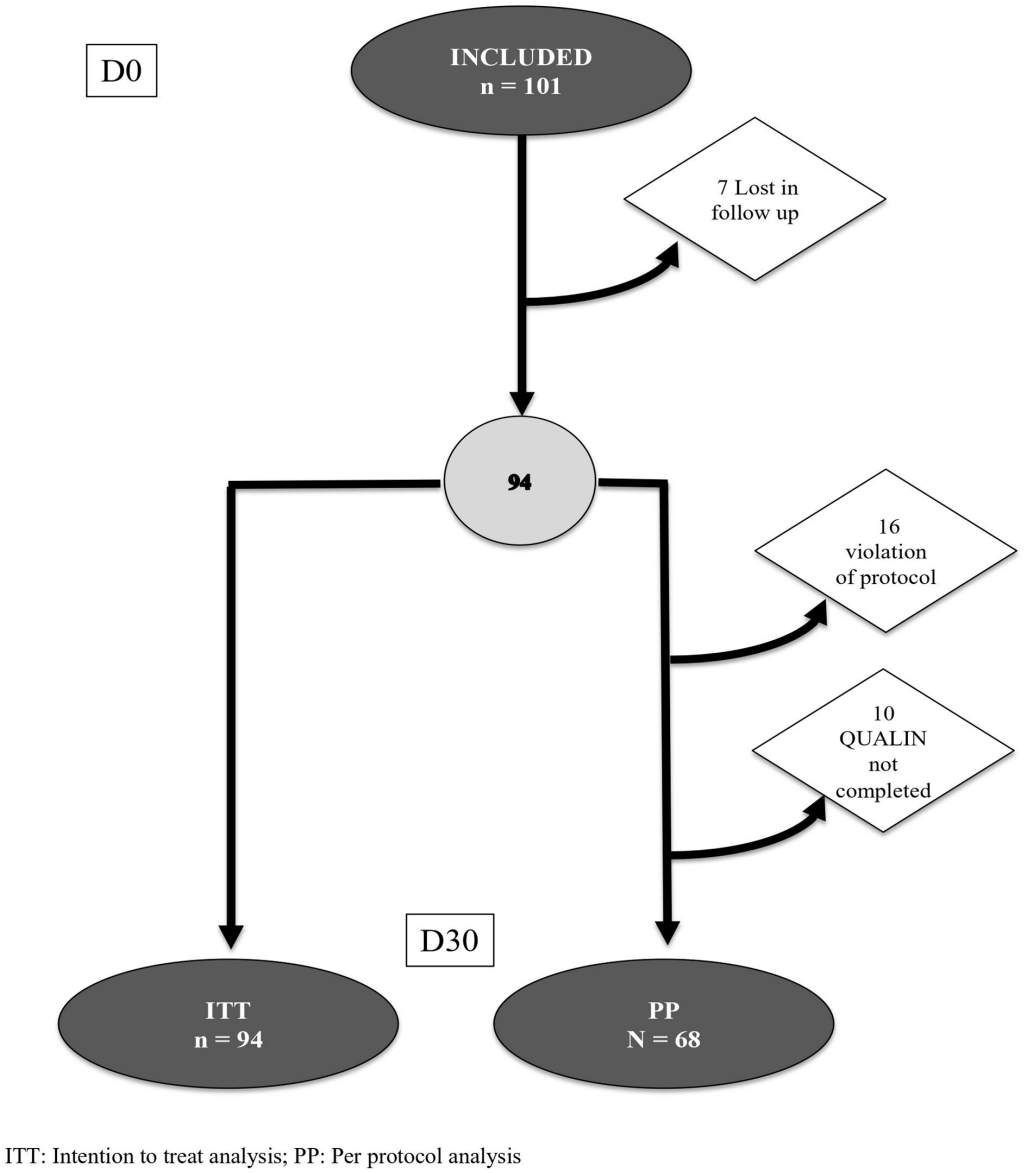
Flow chart.

Of the 68 paediatricians contacted, 53 were initially interested in the study and 28, who were distributed throughout the national territory, finally voluntarily actively participated in the study. Between end of august 2020 and end of October 2021, they included 101 infants presenting regurgitation or excessive crying or both [number of inclusions/pediatrician: mean: 3.6 ± 2.4 (4.0; 2.0–5.0; 1.0–10.0)]. Figure 1 shows the subjects’ flow during the study. Of the included infants, 7 were lost to follow up before D30 (ITT, *n* = 94). For the per protocol analysis (*n* = 68), 16 infants were dropped out because of violation of protocol (3 formula change, 7 drug added, 6 both) and 10 infants because of an uncompleted QUALIN questionnaire.

### Baseline characteristics of the population

They are showed in Table 2, with no difference between the ITT and PP populations or between gender. All infants were born at term, with a normal birth weight. Their median age at inclusion was 5 weeks. Only 13% of infants were older than 3 months, with the oldest one aged 18 weeks. Among included infants, 33 presented with regurgitation only, 34 with colic only and 34 with both regurgitation and colic leading to a sum of prevalence of the two FGIDs over 100%. All the 67 infants with regurgitation had fulfilled the Rome IV criteria (*n* ≥ 2/ day), whereas among the 68 colic infants 95.6% had excessively cried or fussed for more than 3 days a week, but only 22% had presented it during more than 3 h per day and had thus met the full Rome IV criteria (1). According to the Rome IV criteria, five infants could be considered as constipated (≤2 bowel movements/day), and none had a functional diarrhea (≥4 large, unformed stools).

**Table 2:** Baseline characteristics of the population.

	Included	PP
*n*	101	68
Male *n* (%)	50/101 (49.5)	34/68 (50.0)
Rank in siblings	(*n* = 99)	(*n* = 67)
% as no. 1; 2; 3; 4; 5	56.6; 26.3; 15.2; 1.0; 1.0	56.7; 25.4; 17.9; 0.0; 0.0
Gestational age	(*n* = 100)	(*n* =68)
Mean week (SD)	39.3 (1.1)	39.3 (1.1)
Median (*Q*1−*Q*3; range)	39.0 (39.0−40.0; 37.0−41.0)	39.0 (38.0−40.0; 37.0−41.0)
Birth weight	(*n* = 100)	(*n* = 68)
Mean kg (SD)	3.30 (0.40)	3.31 (0.42)
Median (*Q*1−*Q*3; range)	3.33 (3.00−3.50; 2.50−4.34)	3.35 (3.04−3.56; 2.50−4.34)
Birth length	(*n* = 100)	(*n* = 68)
Mean cm (SD)	49.3 (2.1)	49.2 (2.2)
Median (*Q*1−*Q*3; range)	50.0 (48.0−51.0; 44.0−54.0)	50.0 (48.0−51.0; 44.0−54.0)
Age at inclusion	(*n* = 100)	(*n* = 68)
Mean weeks (SD)	6.2 (4.3)	6.3 (4.3)
Median (*Q*1−*Q*3; range)	5.0 (3.0−9.0; 0.3−18.0)	5.0 (3.0−8.0; 0.3−18.0)
Weight	(*n* = 100)	(*n* = 68)
Mean kg (SD)	4.74 (1.11)	4.69 (1.11)
Median (*Q*1−*Q*3; range)	4.45 (3.97−5.30; 2.70−7.80)	4.45 (3.90−5.28; 2.70−7.80)
Length	(*n* = 100)	(*n* = 68)
Mean cm (SD)	55.2 (4.4)	55.0 (4.2)
Median (*Q*1−*Q*3; range)	54.0 (52.0−58.0; 48.0−67.0)	54.0 (52.0−58.0; 48.0−65.0)
Head circumference, cm	(*n* = 100)	(*n* = 68)
Mean cm (SD)	37.8 (2.1)	37.8 (2.1)
Median (*Q*1−*Q*3; range)	37.0 (37.0−39.0; 34.0−44.0)	37.0 (37.0−39.0; 34.0−43.0)
Number (%) of infants with regurgitations	67 (66.4)	42 (61.8)
Number (%) of infants with colic	68 (67.3)	44 (64.7)
Number (%) of infants with both FGIDs	34 (33.7)	18 (26.5)
Number (%) of infants with mostly regurgitation^a^	50 (49.5)	33 (48.5)
Number (%) of infants with mostly colic^a^	46 (45.5)	35 (51.5)
Number (%) of infants with as much regurgitation as colic^a^	5 (5)	0 (0)
Number of bowel movements/week^b^		
Mean (SD)	9.3 (5.3)	9.7 (5.3)
Median (*Q*1−*Q*3; range)	7 (6–14; 1–21)	7 (6–14; 2–21)
Number (%) of infants with hard stool^b^	12 (11.9)	7 (10.3)
Number (%) of infants with normal stool^b^	31 (30.7)	21 (30.9)
Number (%) of infants with smooth or lumpy stool^b^	45 (44.6)	31 (45.6)
Number (%) of infants with curdy or liquid stool^b^	13 (12.9)	9 (13.2)

a According to pediatrician’s assessment.

b As reported by parents during the previous week.

All infants included were fully formula-fed except for one who had started few complementary foods. From birth to D0, 89% of infants were always fed the same formula, 6% had changed formula once, 3% twice, and 2% four times. No infant was fed a formula with all the same characteristics as the study formula. The formula used prior to inclusion were: standard formula (*n* = 1); standard formula with probiotics (*n* = 45), or prebiotics (*n* = 32), or both (*n* = 1); formula with reduced content of lactose only (*n* = 2) or with additionally probiotics and prebiotics (*n* = 13); thickened formula with probiotics (*n* = 5) or with prebiotics (*n* = 2). Moreover 11 infants had received treatment with probiotics, which was LrD in 10. Thus, in total, 55 infants had received LrD before D0, including 45 for whom it was administered as a component of a formula.

On average the final visit (D30) occurred 30.1 ± 2.2 days (30; 29–32; 21–36) after D0.

### Primary outcome

The QoL of 75% of infants in the PP analysis improved at D30 without difference between gender or age, although colicky infants tended to be slightly younger. The mean improvement in the global score was +8.2 ± 13.7 (8; 1–18; −23 to 40; *p* < 0.001) (Table 3). The prevalence of infants with an improved score tended to be higher in the group with only colic (77%) and in the group with colic and regurgitation (89%) than in the group with only regurgitation (62.5%). The QUALIN score significantly increased in infants with colic alone (+9.7 ± 13.8; 10; 1–18; −18 to 40; *p* = 0.001) or in association with regurgitation (+13.3 ± 13.9; 16; 6–24; −15 to 33; *p* < 0.001) and not in infants with only regurgitation (+2.7 ± 11.9; 4; −4 to 10; −23 to 24). This improvement was mainly due to items related to behavior and communication and well-being (66.2% of infants) among which the question related to crying (no. 30) most often got an improved response (54.4% of infants), followed by items no. 5, 13, 21 for about 40% of infants. This group of items accounted, in median value (*Q*1–*Q*3), for 36.4% (28.1–42.5) of the global result at D0 and 37.1% (33.3–42.6) at D30. Worsening of the score was noted in 19% of infants, mainly in the items related to the ability to remain alone (39% of infants), which accounted for less than 5% of the total score. In linear regression, no relationship between the global QoL score and the improvement in the daily number of regurgitations or the weekly number of days with colic was found.

**Table 3:** Evolution of the QUALIN scores from baseline to day 30 according to the symptoms presented in per protocol analysis.

	Infants with regurgitations		Infants with colic		Infants with regurgitations and colic		All infants	
*n*	24	26	18	68	*n*	24	26	18
	D0	D30	D0	D30		D0	D30	D0
Total score								
Mean (SD)	37.7 (9.9)	40.3 (12.2)	29.0 (14.1)	38.7 (10.4)***	30.9 (12.6)	44.3 (8.4)***	32.6 (12.7)	40.8 (10.7)***
Median (*Q*1–*Q*3)	39 (30–47)	43 (34–50)	30 (18–41)	39 (33–47)	28 (20–40)	47 (42–49)	35 (23–42)	44 (35–49)
[Range]	[20–55]	[8–60]	[5–55]	[16–60]	[14–54]	[24–56]	[5–55]	[8–60]
Behavior and communication								
Mean (SD)	13.4 (6.0)	14.6 (5.5)	10.0 (7.4)	14.7 (5.4)	10.9 (7.0)	16.8 (4.6)	11.5 (6.9)	15.2 (5.2)
Median (*Q*1–*Q*3)	14 (9–19)	15 (12–19)	11 (6–16)	16 (12–19)**	11 (5–18)	18 (15–21)**	12 (7–18)	16 (12–20)***
[Range]	[0–22]	[1–22]	[−9 to 22]	[0–22]	[−2 to 21]	[6–22]	[−9 to 22]	[0–22]
Ability to remain alone								
Mean (SD)	1.6 (1.6)	1.7 (1.7)	1.5 (2.0)	1.7 (1.7)	2.1 (1.5)	2.2 (1.4)	1.7 (1.7)	1.8 (1.6)
Median (*Q*1–*Q*3)	2 (1–3)	2 (0–3)	2 (0–3)	2 (0–3)	2 (1–3)	2 (1–3)	2 (1–3)	2 (0–3)
[Range]	[−2 to 4]	[−2 to 5]	[−2 to 5]	[−2 to 5]	[0–5]	[0–4]	[−2 to 5]	[−2 to 5]
Family environment								
Mean (SD)	6.6 (1.4)	6.6 (1.6)	6.1 (1.5)	6.6 (1.4)	6.1 (2.1)	7.0 (1.3)	6.3 (1.6)	6.7 (1.4)
Median (*Q*1–*Q*3)	7 (6–8)	7 (6–8)	6 (5–7)	7 (6–8)	7 (5–8)	8 (6–8)	7 (5–8)	7 (6–8)
[Range]	[4–8]	[4–8]	[2–8]	[4–8]	[2–8]	[4–8]	[2–8]	[4–8]
Psychological and somatic well-being								
Mean (SD)	4.6 (2.5)	6.0 (3.1)	1.2 (4.8)	3.8 (4.0)**	1.5 (4.2)	5.4 (3.0)***	2.5 (4.2)	5.0 (3.5)***
Median (*Q*1–*Q*3)	4 (3–6)	6 (3–9)	0 (−3 to 4)	4 (1–7)	1 (−2 to 4)	7 (2–8)	3 (−1 to 6)	5 (2–8)
[Range]	[−1 to 9]	[0–11]	[−5 to 11]	[−5 to 10]	[−6 to 10]	[2–10]	[−6 to 11]	[−5 to 11]

**p < 0.01, ***p ≤ 0.001.

### Secondary outcomes (regurgitation and colic)

Table 4 shows the different outcomes.

**Table 4:** Outcome of regurgitation or colic from day 0 to day 30 in intention to treat (ITT) and per protocol (PP) analyses.

		Infants with regurgitations		Infants with colic		Infants with regurgitations and colic	
Analysis		ITT	PP	ITT	PP	ITT	PP
*n*		29	24	33	26	32	18
Number of regurgitations/day							
D0	Mean (SD)	5.4 (2.2)	5.0 (2.0)	–	–	4.3 (1.8)	4.4 (1.8)
	Median (*Q*1–*Q*3)	5 (4–6)	5 (3–6)	–	–	4 (3–6)	5 (3–5)
	[Range]	[2–9]	[2–9]	–	–	[2–8]	[2–8]
D30	Mean (SD)	1.8 (1.3)***	1.9 (1.4)***	–	–	1.7 (1.0)***	1.3 (0.6)***
	Median (*Q*1–*Q*3)	1 (1–2)	1 (1–3)	–	–	1 (1–2)	1 (1–2)
	[Range]	[1–5]	[1–5]	–	–	[1–5]	[1–5]
Number of days per week with crying or fussing							
D0	Mean (SD)	–	–	6.0 (1.2)	6.2 (1.0)	5.7 (1.9)	6.3 (1.2)
	Median (*Q*1–*Q*3)	–	–	6 (5–7)	7 (6–7)	7 (4–7)	7 (6–7)
	[Range]	–	–	[3–7]	[3–7]	[1–7]	[4–7]
D30	Mean (SD)	–	–	2.1 (2.0)***	1.7 (1.8)***	2.0 (2.0)***	1.5 (1.6)***
	Median (*Q*1–*Q*3)	–	–	2 (0–3)	2 (0–2)	2 (0–3)	1 (0–2)
	[Range]	–	–	[0–7]	[0–7]	[0–7]	[0–4]
Daily crying duration (mn)							
D0	Mean (SD)	–	–	152.3 (116.2)	150.3 (121.2)	86.7 (68.1)	87.1 (75.4)
	Median (*Q*1–*Q*3)	–	–	120 (90–180)	120 (90–180)	60 (48–120)	60 (50–120)
	[Range]	–	–	[4–600]	[4–600]	[3–300]	[3–600]
D30	Mean (SD)	–	–	39.2 (44.1)***	35.1 (45.8)***	37.0 (49.1)***	19.4 (21.8)***
	Median (*Q*1–*Q*3)	–	–	30 (0–60)	25 (0–55)	20 (5–60)	15 (0–30)
	[Range]	–	–	[0–180]	[0–180]	[0—240]	[0–60]

*p < 0.05, **p < 0.01, ***p < 0.001.

Overall, the daily number of regurgitations decreased from 4.8 ± 2.0 at D0 to 1.7 ± 1.2 at D3O (ITT, *n* = 61; *p* < 0.001), and from 4.7 ± 1.9 to 1.7 ± 1.2 (PP, *n* = 42; *p* < 0.001) in ITT, the decrease was– 3.1 ± 2 (−3; −4; −2; −8 to 2) regurgitations per day, i.e., minus 60.7 ± 26.3% (66.7; 50–80; 20–88). The number of infants in ITT analysis meeting the Rome IV criteria for regurgitation decreased from 100 to 38% at D30. In ITT analysis, 91.8% of parents have noticed a decrease in regurgitation in less than 4 days for 59% of them and within one week for 89%. In PP analysis the decrease in the daily number of regurgitations was of 63.4 ± 17.8% (66.7; 50–80; 20–88); of parents, 98% noticed such an improvement, within one week for 95% of them.

In infants with colic, the weekly number of days with crying or fussing decreased from 5.8 ± 1.5 to 2.0 ± 2.0 in ITT analysis (*n* = 65; *p* < 0.001) and from 6.3 ± 1.1 to 1.5 ± 1.6 in PP analysis (*n* = 44; *p* < 0.001). This corresponded to a decrease of 63.4 ± 37.5% (71; 50–100; 75–100) and 73.6 ± 27.8% (82; 59–100; 0–100) respectively. Of parents 89% (ITT) and 95% (PP) noticed a decrease in number of crying/fussing; it was within 3 days for 36 and 45% and within one week for 76 and 83% of parents, respectively. In PP analysis, parents reported 100% of infants with colic had 3 or more days with crying per week at D0 and 22% at D30. On the other hand, they reported 27% of infants who cried for ≥3 h /day at D0 and 2% at D30. Overall, the cumulative daily crying duration decreased from 120.0 ± 100.5 mn to 38.1 ± 46.3 mn in ITT analysis (*n* = 65; *p* < 0.001), i.e., a decrease of 82 ± 106 mn, and from 124.5 ± 108.5 mn to 28.7 ± 38.3 mn in PP analysis (*n* = 44; *p* < 0.001). Specifically, 90% of the 32 infants with colic who previously to inclusion received LrD, either as drops or in a formula, had improved. In these 32 infants, the number of days per week with colic decreased from 6.1 ± 1.4 to 2.1 ± 2.4 (*p* < 0.001); the median value decreased from 7 (5–7; 2–7) to 1 (0–3; 0–7). In these infants the crying duration per day decreased from 131.4 ± 121.2 mn (90; 60–180; 4–600) to 42.3 ± 51.7 (30; 0–60; 0–240) (*p* < 0.001).

### Evolution of stool frequency and consistency

**Figure 2:**
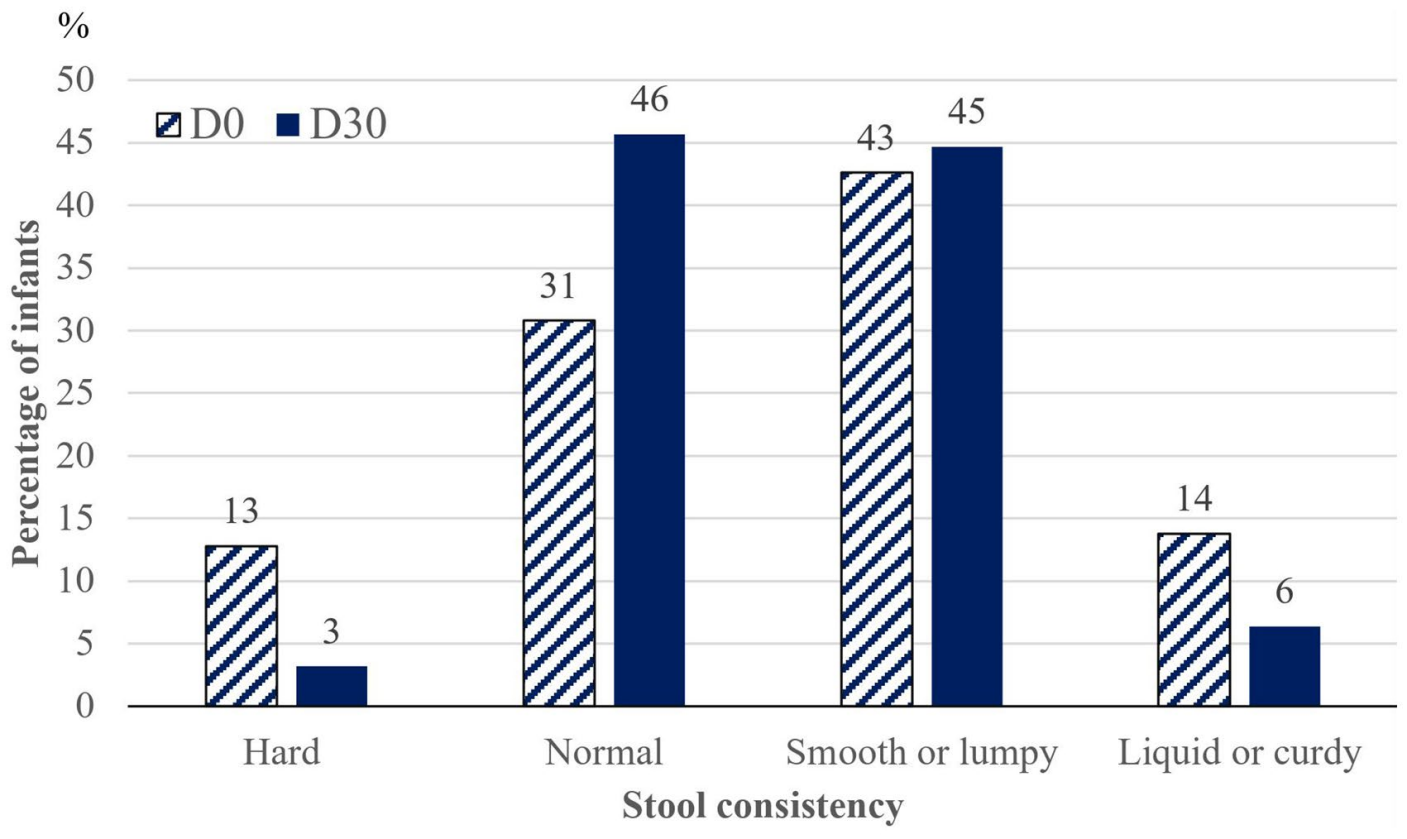
Evolution in stool consistency from inclusion (D0) to endpoint (D30) (*n* = 94). Prevalence of infants with each type of stool.

Little change in the number of bowel movements per week was shown from 9.3 ± 5.3 (7; 6–14; 1–21) at D0 to 8.2 ± 4.0 (7; 6–10; 1–21) at D30. At D30 there was a trend for fewer infants with hard or liquid/curdy stool and for more infants with normal stools, and no more infants who could be considered constipated (Figure 2). These changes in stool consistency were noticed by 45.7% of parents, of whom 41.9% noticed it within 3 days after introduction of the study formula and 80.5% within one week.

### Satisfaction, tolerance and adverse events

Of parents in ITT analysis (*n* = 94) and PP analysis (*n* = 68), 84 and 85% considered that the comfort of the infant had been improved during the study period and 78.7% and 83% asked to continue with the study formula, respectively. Of the 20 and 11 parents who in each analysis did not ask to continue, 13 and 8 considered the formula to be ineffective, and 7 and 3 that the problems were resolved, respectively.

The quantity of formula consumed at D30 was 798.4 ± 99.4 mL/day (750; 720–900; 540–1,080), without difference between infant with regurgitations and infants with colic. Solid foods were introduced in 4 more infants than at D0. The weight gain during the study period in the PP population was 28.8 ± 8.2 g/day (27.1; 23.8–33.4; 11.3–49.7). The length gain was 3.3 ± 1.3 cm (3; 2–4; 1–8) and that of head circumference 1.8 ± 0.8 cm (2; 1–2; 1–5).

Seven adverse events were reported including two constipations (as assessed by parents but not in accordance with Rome IV criteria), two increases in regurgitation, one worsening of colic, one urine tract infection and one gastroenteritis.

## Discussion

This multicenter prospective experimental study was conducted in routine circumstances leading to include infants who did not all meet the Rome IV criteria. Such infants precisely represent the target population concerned by such a dietary approach in current pediatric practice. Such a real-life study is better able to inform on the effectiveness of a management (43). The completion of recruitment has required an unusually relatively long time of over 14 months due to three waves of COVID epidemic (44). The physician’s goal as well as the parents’ expectations in the management of infant’s regurgitations and colic are to improve the comfort of the infant as evidenced by the QoL score by ensuring an alleviation of the symptoms, what were the criteria adopted to judge the effectiveness.

### Effectiveness of the study formula on infants’quality of life and clinical outcome

At inclusion the QoL score in our population of infants with colic or regurgitation was much lower than the one originally reported in healthy 1–3 years children (~53) but higher than that reported in chronic diseases (~23) (41). It is comparable to that already reported in infants with FGIDs (27.2 ± 15.1) (15). In studied infants, the QoL score was lower in those with colic or with both symptoms than in those with regurgitations only, confirming how stressing and depressing the crying/fussing problems could be for parents and for the perception of their infant’s QoL (45). Just over a third of the included infants presented with both FGIDs. Infants presenting multiple FGIDs have already been reported as having a lower QoL score and a slower recovery than those with a single symptom (9).

The results of our study suggest that a starch thickened “comfort formula” containing the probiotic *Limosilactobacillus reuteri* DSM 17938, a mixture of prebiotics FOS/GOS and a reduced content of lactose is effective in the management of infant’s regurgitation and/or colic. The findings were an improvement of the quality of life in three quarters of infants and a decrease in the daily number of regurgitations and in the number of days with colic as well as of daily cumulative duration of crying. The improvement in QoL was more frequently observed in infants with colic, even if they were associated with regurgitations. A greater improvement in QoL score in case of combined FGIDs was previously shown (9). As shown by others, no relationship between the improvement in QoL and that of symptoms could be demonstrated by linear regression (16). The rapid improvement of the symptoms certainly contributes greatly to reassuring parents, what is the cornerstone in the management of these two FGIDs, especially in case of colic (1, 46). It certainly increased “free time” and improved “sleep time” of parents what participated in the perception of better QoL.

Both FGIDs are time-limited conditions. However, age at inclusion (75% of infants were younger than 9 weeks and the oldest included infant was 18 weeks old) and duration of the trial (4 weeks) make the possibility of a natural temporal evolution unlikely to explain the improvements observed (47, 48). The natural evolution of regurgitation shows indeed a peak around 4 months of age and a tapering from 6 months onwards (1, 49, 50). On the other hand, the literature displays substantial variation in the reported age at which the excessive crying stopped, ranging from 9 to 104 weeks (median 19 weeks) with a consensus to consider it after 6 months (1, 9, 46, 51). At the end of our study the oldest infant was 22 weeks old. Therefore, the hypothesis of a formula-specific effect on symptoms outcome seems more than likely in most infants, whereas a spontaneous evolution during the study period seems less likely but cannot be completely excluded in the infants older than 4 months.

The tested formula was well-tolerated and supported adequate infant growth. It has satisfied the vast majority of parents.

### Contribution of the different ingredients to the explanation of the results

Given the diversity of modifications made in the composition of the study formula compared to a standard formula, it is difficult to assess the contribution of each to the observed improvement. Obviously, the thickening of the formula with starch contributed largely to the alleviating of regurgitation as generally admitted (24, 25, 52–55). On the other hand, LrD has been shown to accelerate gastric emptying and improve regurgitation in infants (56). Regarding the improvement in colic, whose etiology remains elusive, our results are consistent with those from studies using formulas also containing LrD but with partially hydrolyzed protein (16, 35, 36). In exclusively breastfed colic infants, the use of LrD is supported by RCTs and meta-analysis, whereas studies in formula fed colic infants are rare and inconsistent and often performed with LrD given as drops and not as an ingredient of the formula (30, 32, 34, 37). In their recent position paper, the ESPGHAN Special Interest Group on Gut Microbiota and Modifications considered that no recommendation could be made so far for or against its use in colic formula fed infants due to insufficient evidence (32). However, the group stated that LrD as well as *B. lactis* BB-12 may be recommended for the management of colic in breastfed infant. The rationality of using LrD is based on the demonstrated notion of an imbalanced microbiota colonization in infants with colic (11, 57, 58). A low intestinal concentration of lactobacilli genera would have an important role in the pathophysiology of infantile colic and, on the other hand, LrD would reduce inflammation, gas production, and pain perception (37). Besides this probiotic, the study formula, like the one used by Vandenplas et al. with partially hydrolyzed protein, contained FOS/GOS leading to consider the possibility of a synbiotic effect (16), as defined by the ISAPP consensus statement (31). This might participate in explaining the improvement of infants having previously received LrD but then without effectiveness. In addition, a fermented formula for which the bacterial fermentation process is followed by mild heat treatment and that contained FOS/GOS has been shown to be more effective in preventing infant’s colic than the same formula without FOS/GOS or an unfermented formula containing FOS/GOS (59). The role of excess lactose in the onset of colic has been questioned and related to a transient low lactase activity in young infants (27). The undigested lactose then reaches the colon where its bacterial fermentation produces gas including hydrogen, and intestinal distension that possibly triggers crying (11). Greater baseline breath hydrogen excretion at baseline as well as after a lactose meal have been reported in some infants with colic compared with healthy infants, but this may have been contradicted by others (27, 60). Randomized clinical trials of oral lactase administration as well as trials with reduced lactose content have shown conflicting results in the management of infantile colic (27, 60–62). A reduced lactose content (5.0 g/100 mL) in the study formula may thus have contributed to alleviation of crying. In total, the combination of all the changes made to the formula studied is presumably at the origin of the results observed, without it being possible to formally conclude on the interest of each of them. This would require randomized studies comparing formulas with an isolated modification and then combining them in different ways. Such studies are almost impossible.

## Strengths and limitations

The strength of our study is that effectiveness assessment was based on both clinical outcome and QoL as recommended by Rome IV consensus (1). Of included infants only 7% were lost to follow-up and full adherence to protocol was observed in more than 67% of parents. The main advantage of such a pragmatic real-life study is its natural practice setting, mimicking every day clinical practice which provides high external validity (43). The possibility of having an overestimation of the number of regurgitations or of the amounts of crying by the parents is counterbalanced by the fact that the evaluation of their evolution was carried out within the subject in a pre- post-test.

Some limitations must be acknowledged. The report of data by parents lead to a few rare outliers that sometimes constitute the extreme data of the ranges. Some questions of the QUALIN questionnaire might not be well suited to very young infants or may have embarrassed some parents, which could explain the number of uncompleted questionnaires. Finally, the absence of randomization with a control group leads to consider the possibility of a placebo effect. Colic was shown to be highly responsive to placebo in different studies (63, 64). The parents’ awareness of the potential effect of the formula as well as the reassurance measures provided by the pediatrician probably interacted in the improvements observed (13, 37, 46). However, the placebo and reassurance effects are components of the response to treatment normally present in clinical practice and were certainly acting before inclusion, especially since 11% of infants had previously changed from formula, 68% had already received a probiotic, and 45% prebiotics, but without effectiveness. This leads to the conclusion that the study formula is effective as a whole.

## Conclusion

This study shows that, in routine clinical practice, a starched thickened formula with reduced lactose content and supplemented with *Limosilactobacillus reuteri* DSM 17938 and a mixture of fructo-oligosaccharides and galacto-oligosaccharides has more than likely helped improve quality of life of young infants with regurgitation or colic or both and alleviate the underlying symptoms. As a consequence, such a formula deserves to be prescribed, in association with reassurance to parents, in the management of these infants without waiting for a possible spontaneous improvement which can be much later. On the other hand, the study confirms the absolute non-necessity of drugs in the management of these FGIDs. However, none of the specific ingredients of the test formula can be directly associated with the observed improvement, but the formula as a whole, unless separate RCTs are carried out in the future with each of them.

## Data availability statement

The raw data supporting the conclusions of this article will be made available by the authors, without undue reservation.

## Ethics statement

The studies involving human participants were reviewed and approved by Subject Protection Review Board “Sud Mediterrannée III” (Nimes 2020/03/18, no. 2020.01.07 bis_19.12.26.60314). Written informed consent to participate in this study was provided by the participants’ legal guardian/next of kin.

## Author contributions

J-PC, SB, BD, and TH contributed equally to the conception and design of the study, as well as analysis, and interpretation of data; they had full access to all the data in the study and take responsibility for the integrity of the data and the accuracy of the data analysis. J-PC wrote the original draft. SB, BD, and TH revised it critically. All authors contributed to the article and approved the submitted version.

## Funding

This work was funded by Nestlé France, Département Médical Nutrition Infantile, Issy-les-Moulineaux, France.

## Conflict of interest

J-PC served as consultant for this study. SB received honoraria for her contribution to the conduct of the study, fees from Bledina for writing a leaflet and from the AFPA for expert testimony. BD and TH are employees of Nestlé France.

The authors declare that this study received funding from Nestlé. The funder had the following involvement in the study: participation in the study design and in reviewing the final manuscript to approve it without involvement in data analysis, discussion nor conclusion.

## Publisher’s note

All claims expressed in this article are solely those of the authors and do not necessarily represent those of their affiliated organizations, or those of the publisher, the editors and the reviewers. Any product that may be evaluated in this article, or claim that may be made by its manufacturer, is not guaranteed or endorsed by the publisher.

## Abbreviations

FGIDs: Functional gastrointestinal disorders
FOS: Fructo-oligosaccharides
GOS: Galacto-oligosaccharides
ITT: Intention to treat
LrD: *Limosilactobacillus reuteri* DSM 17938
PP: Per protocol
QoL: Quality of life
