# Supplementary material for: Effectiveness of a starch thickened infant formula with reduced lactose content, probiotics and prebiotics on quality of life and clinical outcome in infants with regurgitation and/or colic
Source: Front Nutr. 2023 May 25;10:1164722. doi: 10.3389/fnut.2023.1164722 (PMC10249472; doi:10.3389/fnut.2023.1164722)
Supplement: Supplementary file 1 [file Table_1.DOCX]

English version of the QUALIN questionnaire for infant (translated from ref 41)

| **Quality of Life scoring** | | | | | | |
| --- | --- | --- | --- | --- | --- | --- |
| **Items** | Definitely false | Mostly false | Both true and false | Mostly true | Definitely true | I do not know |
| 1- He/she eats well | 🞎 | 🞎 | 🞎 | 🞎 | 🞎 | 🞎 |
| 2- He/she has good-looking | 🞎 | 🞎 | 🞎 | 🞎 | 🞎 | 🞎 |
| 3- He/she is lively | 🞎 | 🞎 | 🞎 | 🞎 | 🞎 | 🞎 |
| 4- He/she is often painful somewhere | 🞎 | 🞎 | 🞎 | 🞎 | 🞎 | 🞎 |
| 5- He/she plays willingly | 🞎 | 🞎 | 🞎 | 🞎 | 🞎 | 🞎 |
| 6- He/she is restless | 🞎 | 🞎 | 🞎 | 🞎 | 🞎 | 🞎 |
| 7- He/she likes to be looked after | 🞎 | 🞎 | 🞎 | 🞎 | 🞎 | 🞎 |
| 8- He/she is happy, laughs or smiles easily | 🞎 | 🞎 | 🞎 | 🞎 | 🞎 | 🞎 |
| 9- He/she goes easily to others | 🞎 | 🞎 | 🞎 | 🞎 | 🞎 | 🞎 |
| 10- He/she always needs to be reassured | 🞎 | 🞎 | 🞎 | 🞎 | 🞎 | 🞎 |
| 11- He/she has a good family environment | 🞎 | 🞎 | 🞎 | 🞎 | 🞎 | 🞎 |
| 12- He/she is developing well and is healthy | 🞎 | 🞎 | 🞎 | 🞎 | 🞎 | 🞎 |
| 13- He/she is playful, mischievous | 🞎 | 🞎 | 🞎 | 🞎 | 🞎 | 🞎 |
| 14- He/she often seems worried | 🞎 | 🞎 | 🞎 | 🞎 | 🞎 | 🞎 |
| 15- He/she is trying to get attention | 🞎 | 🞎 | 🞎 | 🞎 | 🞎 | 🞎 |
| 16- He/she is full of energy and life | 🞎 | 🞎 | 🞎 | 🞎 | 🞎 | 🞎 |
| 17- He/she cries as soon as he/she feels alone | 🞎 | 🞎 | 🞎 | 🞎 | 🞎 | 🞎 |
| 18- He/she likes playing | 🞎 | 🞎 | 🞎 | 🞎 | 🞎 | 🞎 |
| 19- He/she adapts easily to changes | 🞎 | 🞎 | 🞎 | 🞎 | 🞎 | 🞎 |
| 20- He/she is tiresome | 🞎 | 🞎 | 🞎 | 🞎 | 🞎 | 🞎 |
| 21- He/she is chirping well | 🞎 | 🞎 | 🞎 | 🞎 | 🞎 | 🞎 |
| 22- He/she is interested in everything and open to others | 🞎 | 🞎 | 🞎 | 🞎 | 🞎 | 🞎 |
| 23- He/she often asks to be carried | 🞎 | 🞎 | 🞎 | 🞎 | 🞎 | 🞎 |
| 24- He/she is graceful | 🞎 | 🞎 | 🞎 | 🞎 | 🞎 | 🞎 |
| 25-He/she likes contact with people | 🞎 | 🞎 | 🞎 | 🞎 | 🞎 | 🞎 |
| 26- His/her parents get along well | 🞎 | 🞎 | 🞎 | 🞎 | 🞎 | 🞎 |
| 27- He/she is often ill | 🞎 | 🞎 | 🞎 | 🞎 | 🞎 | 🞎 |
| 28- He/she sleeps well | 🞎 | 🞎 | 🞎 | 🞎 | 🞎 | 🞎 |
| 29- He/she has a regular lifestyle | 🞎 | 🞎 | 🞎 | 🞎 | 🞎 | 🞎 |
| 30- He/she often cries | 🞎 | 🞎 | 🞎 | 🞎 | 🞎 | 🞎 |
| 31- His/her mode of care is satisfactory | 🞎 | 🞎 | 🞎 | 🞎 | 🞎 | 🞎 |
| 32- He/she is coaxing, affectionate | 🞎 | 🞎 | 🞎 | 🞎 | 🞎 | 🞎 |
| 33- When he/she is crying, we understand why | 🞎 | 🞎 | 🞎 | 🞎 | 🞎 | 🞎 |
| 34- He/she is not wild | 🞎 | 🞎 | 🞎 | 🞎 | 🞎 | 🞎 |
